# Primary Gastrointestinal Diffuse Large B-cell Lymphoma Presenting as Ileal Perforation

**DOI:** 10.7759/cureus.37341

**Published:** 2023-04-09

**Authors:** Sean Rumney, Aashish Rajesh, Erika Brigmon

**Affiliations:** 1 General Surgery, University of Texas Health Science Center at San Antonio, San Antonio, USA; 2 Surgery, University of Texas Health Science Center at San Antonio, San Antonio, USA

**Keywords:** primary gastrointestinal lymphoma, non-hodgkin's lymphoma, secondary hemophagocytic lymphohistiocytosis (hlh), ileal perforation, diffuse large b lymphoma

## Abstract

Diffuse large B-cell lymphoma (DLBCL) is the most common form of non-Hodgkin’s lymphoma and can rarely present as a primary gastrointestinal malignancy. Primary gastrointestinal lymphoma (PGIL) is associated with a significant risk of perforation and peritonitis, with high rates of mortality. Here we describe a case of a newly diagnosed PGIL in a previously healthy 22-year-old male presenting for new-onset abdominal pain with diarrhea. Early hospital course was characterized by peritonitis and severe septic shock. Despite multiple surgical interventions and resuscitative efforts, the patient’s condition continued to deteriorate until cardiac arrest and death on hospital day five. A diagnosis of DLBCL of the terminal ileum and cecum was made by pathology post-mortem. The prognosis for these patients can be improved through early intervention with chemotherapy regimens and surgical resection of the malignant tissue. This report highlights DLBCL as a rare cause of gastrointestinal perforation that can culminate in precipitous multiorgan failure and death.

## Introduction

Primary gastrointestinal lymphoma (PGIL) is a rare form of gastrointestinal malignancy with a high associated mortality (about 29% with five-year survival) [1]. In patients presenting with localized abdominal pain and other non-specific GI symptoms the diagnosis of lymphoma relies heavily on imaging studies and, ultimately, pathological review of the tissue in question. In this case report, we discussed the clinical course of a patient presenting with perforation and peritonitis secondary to diffuse large B-cell lymphoma of the terminal ileum and cecum.

## Case presentation

**Figure 1:**
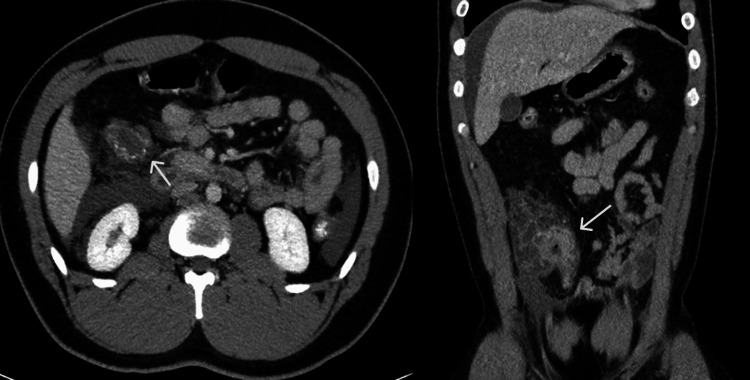
Axial and coronal CT images of the abdomen and pelvis. Long segment of cecum/ascending colon that demonstrates mural thickening, mucosal hyperenhancement, and significant adjacent fat stranding, with multiple local enlarged lymph nodes.

A 22-year-old healthy African-American male presented with chief complaints of two weeks of diarrhea and two days of right lower quadrant (RLQ) abdominal pain, nausea, and vomiting. Examination was notable for RLQ tenderness without peritonitis. Laboratory tests revealed leukocytosis (16.51×10^9^/L) and polycythemia (hemoglobin {Hb} of 19.0 g/dL). Computed tomography (CT) revealed focal inflammatory changes of the terminal ileum (TI) with mucosal hyperenhancement and cecum with small ascites without signs of perforation (Figure 1). The patient was admitted for management and further workup of possible inflammatory bowel disease.

**Figure 2:**
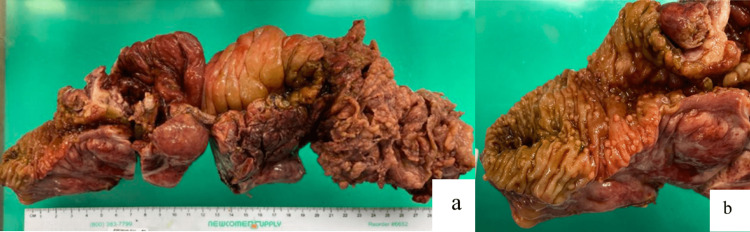
Gross pathology images. The images show (a) ileal ulceration with abscess formation and (b) terminal ileum and long segment of the cecum/ascending colon with mural thickening, mucosal hyperenhancement, and significant adjacent fat stranding, with multiple enlarged lymph nodes. Multiple pseudo-polyps are present at the terminal ileum.

On hospital day two, he developed tachycardia, worsening leukocytosis (41×10^9^/L), lactic acidosis (lactate 7.3 mmol/L), and peritonitis with guarding and rigidity on examination. Exploratory laparotomy revealed an RLQ phlegmon with severe terminal ileal thickening (Figures 2a, 2b). A right hemicolectomy with end ileostomy was performed (proximal end of transverse colon was left stapled off intraabdominally). An anastomosis was not pursued due to hemodynamic instability and vasopressor requirement. Frozen section pathology revealed an ileal ulcer with abscess.

**Figure 3:**
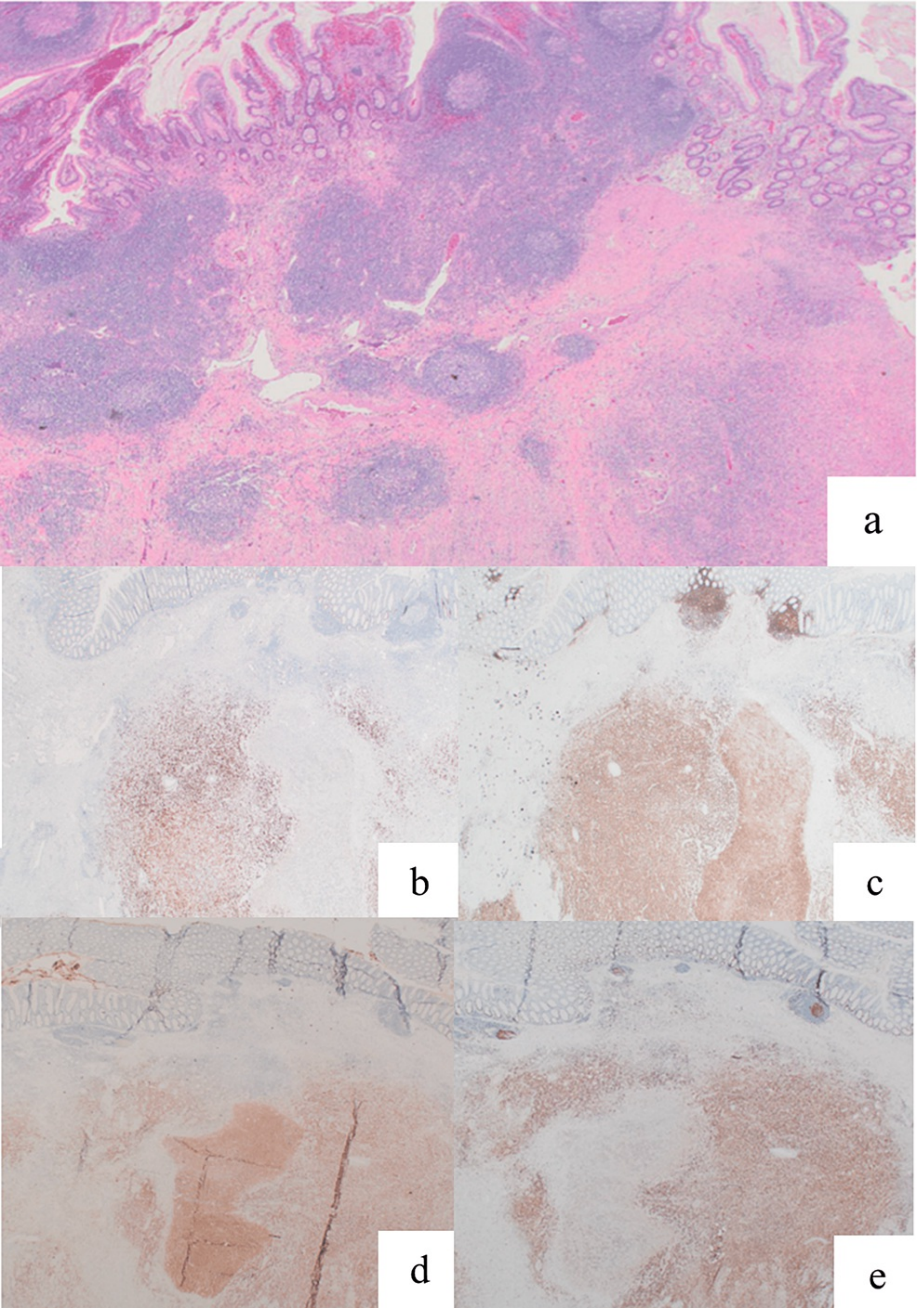
Histology demonstrating evidence of diffuse large B-cell lymphoma. (a) Histological sections show diffuse intramural sheets of atypical, medium- to large-sized lymphoid cells with irregular, hyperchromatic nuclei, prominent basophilic nucleoli, and moderate amounts of amphophilic cytoplasm. There was extensive admixed tumor necrosis with apoptotic debris. (b) Immunohistochemical staining shows large cells are positive for c-MYC (greater than 40% of the cells). (c) Immunohistochemical staining shows large cells are diffusely positive for cluster of differentiation (CD)20. (d) Immunohistochemical staining showed large cells are diffusely positive for CD10. (e) Immunohistochemical staining showed Ki-67. The proliferation index is approximately 70%.

On post-operative day 1, he developed fever, persistent lactic acidosis, and required increasing vasopressor support. Additional workup, including surgical re-exploration, was negative for uncontrolled source of sepsis. Re-exploration was performed due to the lack of other obvious sources of sepsis, as well as out of concern for additional necrotic bowel, given the appearance of the TI at the time of the index operation. The patient's clinical course progressed to disseminated intravascular coagulopathy, hemothorax, rhabdomyolysis, portal vein thrombosis, liver failure (aspartate aminotransferase {AST}: 4,344 U/L, alanine transaminase {ALT}: 1,163 U/L, total bilirubin: 7.2 mg/dL), elevated LDH (5,238 U/L), ferritin (4,969 ng/mL) and acute renal failure with refractory hyperkalemia while on continuous renal replacement therapy leading to cardiac arrest. Post-mortem examination confirmed pulmonary edema, hepatic infarction, and acute ischemic neuronal injury. Final pathology of the surgical specimen revealed an aggressive variant of perforated diffuse high-grade large B-cell lymphoma (DLBCL) without adjacent lymph node involvement (0/28) (Figures 3a-3e).

## Discussion

DLBCL is the most common form of non-Hodgkin’s lymphoma (NHL) and the gastrointestinal (GI) tract is the most common extra-nodal site of involvement. Primary gastrointestinal lymphoma is rare, accounting for 1-4% of GI malignancies [2]. Perforation is associated with a poor prognosis in patients with PGIL, with a decrease in five-year survival from 21.2% to 11.8% in one retrospective study [3]. Female gender, B-cell phenotype, and radical surgery have been found to be associated with a better prognosis in these patients.

A large retrospective review identified 92 perforations out of 1,062 cases of GI lymphoma over a 38-year timeframe. In cases of perforation, DLBCL was found to be the most common histopathology with a 28% mortality rate [4]. Our hypothesis is that our patient developed a hyperinflammatory state that likely triggered a secondary hemophagocytic lymphohistiocytosis (HLH) progressing to multiorgan failure and ultimately causing his death. The diagnosis of secondary HLH is often one of exclusion, and extensive workup for other causes including adrenal insufficiency, pulmonary embolism, and other forms of refractory shock were negative in this patient.

Malignancy-associated HLH has a worse prognosis and survival in these patients has been shown to be poor across multiple studies [5,6]. Rapid clinical deterioration can occur in such patients, and in the proper clinical setting literature supports aggressive treatment with systemic chemotherapy to manage secondary HLH [7-11]. Our patient received steroids during his ICU course but had not received induction chemotherapy as a malignancy diagnosis was only made post-mortem. It is unclear whether chemotherapy would have altered this patient's outcome, but an early multidisciplinary discussion with the oncology team and weighing risk-benefit ratios could have been pursued in the setting of a timely diagnosis.

## Conclusions

This case highlights an unusual etiology of intestinal perforation in a patient presenting with a contained ileal perforation and rapidly progressive sepsis. Patients with intestinal perforation are managed by surgical teams who rarely deal with hematological malignancies, in the appropriate clinical context. Awareness of this entity is of paramount importance to facilitate early consideration for chemotherapeutic regimens that may favorably alter clinical outcomes.
